# Supplementary material for: Cus2 enforces the first ATP-dependent step of splicing by binding to yeast SF3b1 through a UHM–ULM interaction
Source: RNA. 2019 Aug;25(8):1020–37. doi: 10.1261/rna.070649.119 (PMC6633205; doi:10.1261/rna.070649.119)
Supplement: Supplemental Material [file supp_25_8_1020__index.html]

Cus2 enforces the first ATP-dependent step of splicing by binding to yeast SF3b1 through a UHM–ULM interaction — Supplemental Material 

# Cus2 enforces the first ATP-dependent step of splicing by binding to yeast SF3b1 through a UHM–ULM interaction

## Supplemental Material

- Supplemental\_Figure\_S1.tiff
- Supplemental\_Figure\_S2.tif
- Supplemental\_Figure\_Legends.docx
