# Supplementary figures and images for: Cus2 enforces the first ATP-dependent step of splicing by binding to yeast SF3b1 through a UHM–ULM interaction
Source: RNA. 2019 Aug;25(8):1020–37. doi: 10.1261/rna.070649.119 (PMC6633205; doi:10.1261/rna.070649.119)

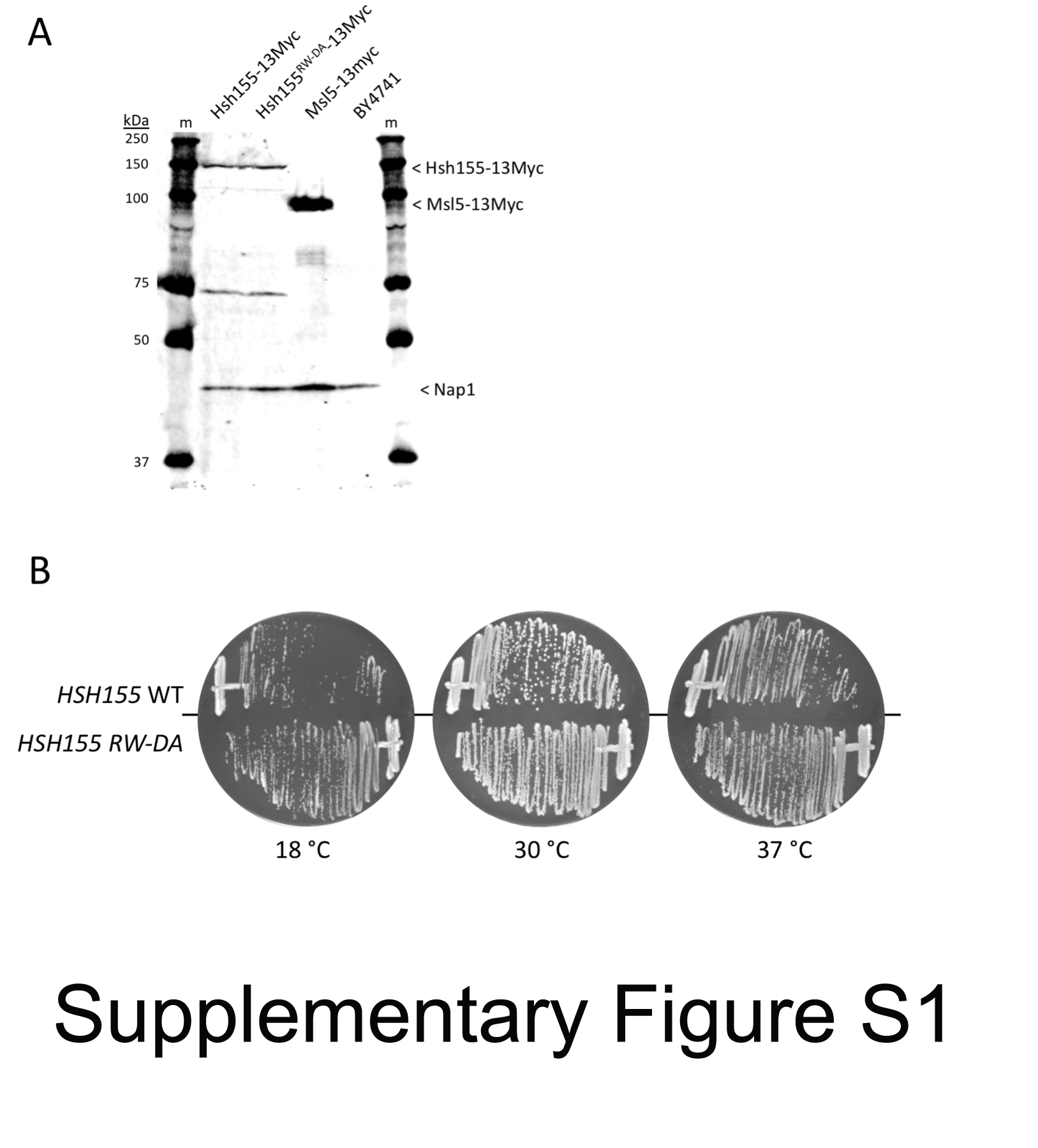

Supplement: Supplemental Material [file supp_070649.119_Supplemental_Figure_S1.tif]

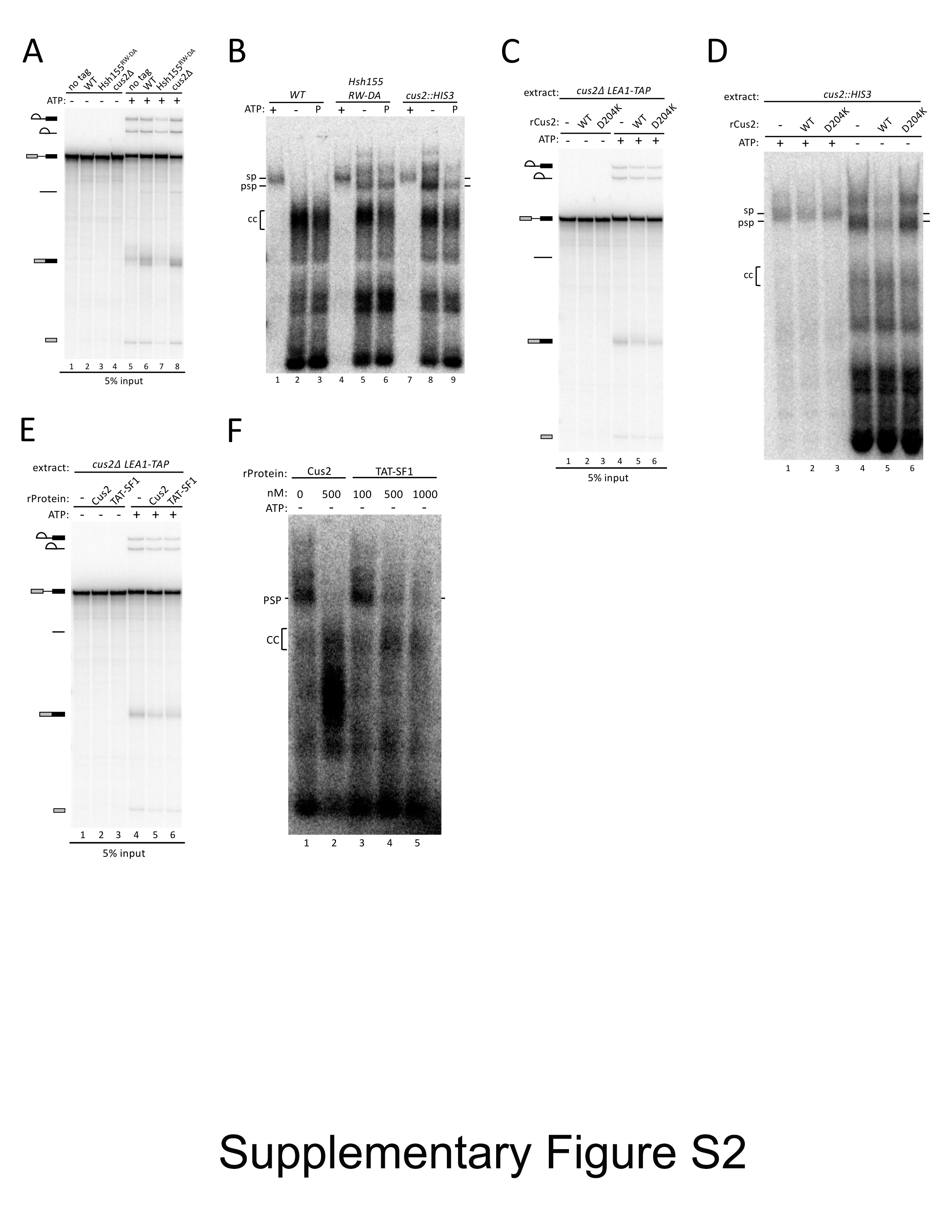

Supplement: Supplemental Material [file supp_070649.119_Supplemental_Figure_S2.tif]
