# Supplementary material for: Cus2 enforces the first ATP-dependent step of splicing by binding to yeast SF3b1 through a UHM–ULM interaction
Source: RNA. 2019 Aug;25(8):1020–37. doi: 10.1261/rna.070649.119 (PMC6633205; doi:10.1261/rna.070649.119)
Supplement: Supplemental Material [file supp_070649.119_Supplemental_Figure_Legends.docx]

**Supplemental Figure S1 (to Figure 5A):** Mutation of the ULM of Hsh155 does not affect protein expression or yeast viability. **(A)** Whole cell extracts prepared from cells expressing wild-type or RW-DA mutant Hsh155-13Myc were resolved by SDS-PAGE and assayed by western blotting. An extract containing Msl5-13Myc serves as a control for the specificity of the mouse anti-Myc antibody. Nap1 serves as a loading control. **(B)** Wild-type and Hsh155^RW-DA^ yeast strains grown at 18 °C (5 days), 30 °C, and 37 °C (2 days) on YEPD medium.

**Supplemental Figure S2 (to Figure 5B-D): (A,C,E)** 5% of the *in vitro* splicing reactions immunoprecipitated in Figures 5B-D. **(B,D,E)** Native gel analysis of spliceosome assembly for reactions described in Figure 5B-D. Pre-RP51A was used as a substrate at a final concentration of 0.4 nM. Spliceosomes (sp), pre-spliceosomes (psp), and commitment complexes (cc) are indicated. **(G)** Co-immunoprecipitation of ATP-independent splicing complex with Lea1-TAP in cus2Δ extracts reconstituted with wild-type and UHM-mutant Cus2 and Tat-SF1 recombinant proteins. The final concentration of recombinant protein in the splicing reaction is indicated in nM. Tat-SF1 “prep 1” and “prep 2” indicate two independent purifications of recombinant Tat-SF1.
